# PA-6 inhibits inward rectifier currents carried by V93I and D172N gain-of-function K_IR_2.1 channels, but increases channel protein expression

**DOI:** 10.1186/s12929-017-0352-x

**Published:** 2017-07-15

**Authors:** Yuan Ji, Marlieke G. Veldhuis, Jantien Zandvoort, Fee L. Romunde, Marien J. C. Houtman, Karen Duran, Gijs van Haaften, Eva-Maria Zangerl-Plessl, Hiroki Takanari, Anna Stary-Weinzinger, Marcel A. G. van der Heyden

**Affiliations:** 1 Department of Medical Physiology, Division of Heart and Lungs, University Medical Center Utrecht, Yalelaan 50, 3584 CM Utrecht, The Netherlands; 2 Center for Molecular Medicine, Department of Medical Genetics, University Medical Center Utrecht, Utrecht, The Netherlands; 3 Department of Pharmacology and Toxicology, University of Vienna, Vienna, Austria

**Keywords:** I_K1_, K_IR_2.1, Atrial fibrillation, Short QT syndrome, Drugs, PA-6, Trafficking

## Abstract

**Background:**

The inward rectifier potassium current I_K1_ contributes to a stable resting membrane potential and phase 3 repolarization of the cardiac action potential. *KCNJ2* gain-of-function mutations V93I and D172N associate with increased I_K1_, short QT syndrome type 3 and congenital atrial fibrillation. Pentamidine-Analogue 6 (PA-6) is an efficient (IC_50_ = 14 nM with inside-out patch clamp methodology) and specific I_K1_ inhibitor that interacts with the cytoplasmic pore region of the K_IR_2.1 ion channel, encoded by *KCNJ2*. At 10 μM, PA-6 increases wild-type (WT) K_IR_2.1 expression in HEK293T cells upon chronic treatment. We hypothesized that PA-6 will interact with and inhibit V93I and D172N K_IR_2.1 channels, whereas impact on channel expression at the plasma membrane requires higher concentrations.

**Methods:**

Molecular modelling was performed with the human K_IR_2.1 closed state homology model using FlexX. WT and mutant K_IR_2.1 channels were expressed in HEK293 cells. Patch-clamp single cell electrophysiology measurements were performed in the whole cell and inside-out mode of the patch clamp method. K_IR_2.1 expression level and localization were determined by western blot analysis and immunofluorescence microscopy, respectively.

**Results:**

PA-6 docking in the V93I/D172N double mutant homology model of K_IR_2.1 demonstrated that mutations and drug-binding site are >30 Å apart. PA-6 inhibited WT and V93I outward currents with similar potency (IC_50_ = 35.5 and 43.6 nM at +50 mV for WT and V93I), whereas D172N currents were less sensitive (IC_50_ = 128.9 nM at +50 mV) using inside-out patch-clamp electrophysiology. In whole cell mode, 1 μM of PA-6 inhibited outward I_K1_ at −50 mV by 28 ± 36%, 18 ± 20% and 10 ± 6%, for WT, V93I and D172N channels respectively. Western blot analysis demonstrated that PA-6 (5 μM, 24 h) increased K_IR_2.1 expression levels of WT (6.3 ± 1.5 fold), and V93I (3.9 ± 0.9) and D172N (4.8 ± 2.0) mutants. Immunofluorescent microscopy demonstrated dose-dependent intracellular K_IR_2.1 accumulation following chronic PA-6 application (24 h, 1 and 5 μM).

**Conclusions:**

1) *KCNJ2* gain-of-function mutations V93I and D172N in the K_IR_2.1 ion channel do not impair PA-6 mediated inhibition of I_K1_, 2) PA-6 elevates K_IR_2.1 protein expression and induces intracellular K_IR_2.1 accumulation, 3) PA-6 is a strong candidate for further preclinical evaluation in treatment of congenital SQT3 and AF.

## Background

In the heart, inward rectifier potassium currents (I_K1_) contribute to stabilization of the resting membrane potential of contractile cardiomyocytes and participate in the final phase of repolarization of the action potential [1]. Gain-of-function mutations in the *KCNJ2* gene, that encodes K_IR_2.1 protein underlying I_K1_, associate with ventricular (short QT syndrome type 3 (SQT3)) and atrial (congenital atrial fibrillation (AF)) phenotypes. D172N and K346T are linked to SQT3, whereas V93I associates with congenital AF [2–4]. E299V and M301K have been linked to both SQT3 and AF [5, 6].

Congenital SQT syndrome is diagnosed in the presence of a QTc interval equal or less than 330 ms, and may be diagnosed at a QTc of less than 360 ms when other conditions apply, like a pathologic mutation or a family history of SQT [7]. Congenital SQT can either be caused to excessive repolarization capacity (SQT1-3), or due to decreased depolarization capacity (SQT4-7), and is associated with high risk for sudden cardiac death and therefore implantable cardioverter-defibrillator (ICD) implantation is indicated [8, 9]. However, pharmacotherapy may be beneficial in patients that are unsuitable for ICD therapy (e.g. young children), those that refuse ICD implantation or for bridging the time to ICD implantation [10]. Some drugs are indeed able to inhibit currents produced by K_v_11.1, K_V_7.1 and K_IR_2.1 channels bearing gain-of-function mutations associated with SQT1, SQT2 and SQT3, respectively [11–14].

AF is associated with increased risk for stroke and heart failure [15]. Action potential lengthening drugs, e.g. targeting the delayed rectifier (I_Kr_), or drugs increasing atrial fibrillation cycle length (sodium current (I_Na_) blockers), have the potential to counteract AF [16]. Inhibition of the acetylcholine activated inward rectifier potassium current (I_KAch_) channel, closely related to the I_K1_ channel, has been proposed as an effective treatment in AF [17]. Also I_K1_ inhibiting compounds, like chloroquine, display anti-AF activity in animal models [18, 19].

We have developed a new I_K1_ inhibiting compound, named PA-6, recently [20]. After crossing the plasma membrane, PA-6 can enter the I_K1_ channel from the cytoplasmic side, will bind to the channel by lipophilic interactions and hydrogen bonds to residues E224, D259 and E299, and subsequently inhibits inward and outward potassium current with an IC_50_ in the low nanomolar range [20]. Recently, we demonstrated that PA-6 lengthens action potential duration, atrial fibrillation cycle length and cardioverts goats with rapid pacing induced AF to sinus rhythm [20, 21]. Interestingly, some ion channel inhibitors are able to increase channel expression [20, 22], or restore normal plasma membrane expression of trafficking defective mutant channels [23–25], probably by stabilizing the channel structure as a result of their direct interaction. Also PA-6 is able to increase expression of wild-type (WT) K_IR_2.1 channels [20].

We hypothesized that PA-6 inhibits I_K1_ channels that are formed by gain-of-function K_IR_2.1 channel proteins and thus can be considered as a candidate drug in treating SQT3 and congenital AF.

## Methods

### Molecular modelling

Docking of compound PA-6 was conducted using the previously constructed closed state homology model of the human K_IR_2.1 channel [20]. In silico mutations of residues V93I and D172N were generated with SwissPdbViewer [26]. Compound PA-6 was generated as described previously [20]. The docking program FlexX (part of the LeadIT software package version 2.0.1 (BioSolveIT GmbH, St Augustin, Germany) was used for docking. The binding site was specified selecting the carboxylic acids of the Glu224 residues from all four subunits. The radius of the binding site was set to 20 Å. Default settings of FlexX were applied for protonation and torsion angles. The ChemScore scoring function of FlexX was applied and the top 10 docking solutions were saved for analysis.

### KCNJ2 constructs

Mutations V93I and D172N were engineered into a human *KCNJ2-*pcDNA3 expression construct [27], using the QuikChange II XL Site-Directed Mutagenesis Kit (Stratagene, La Jolla, CA) and custom designed primers. The presence of the introduced mutations was confirmed by Sanger sequencing.

### Patch-clamp electrophysiology

HEK293T cells were transfected with WT, V93I or D172N constructs together with a GFP expression construct to enable detection of transfected cells. Inside-out patch clamp measurements were made using a HEKA EPC-10 Double Plus amplifier controlled by PatchMaster 2.10 software (HEKA, Lambrecht/Pfalz, Germany) at 21 °C. To record WT, V93I and D172N K_IR_2.1 currents, inside-out patch-clamp measurements were performed using a ramp protocol ranging from −100 to +100 mV in 5 s from a holding potential of −40 mV. Excised patches were placed in close proximity to the inflow region of the perfusion chamber. Bath solution contained 125 mM KCl, 4 mM EDTA (2K), 7.2 mM K_2_HPO_4_, 2.8 mM KH_2_PO_4_, pH 7.20/KOH. Pipette solution contained 145 mM KCl, 1 mM CaCl_2_, 5 mM HEPES, pH 7.40/KOH. Measurements were started following washout of polyamines/Mg^2+^ from the channel pore observed by the disappearance of current rectification. For IC_50_ curves, fractional block at −80 and +50 mV was determined by dividing current levels obtained with PA-6 containing solutions by current levels of control traces recorded in the absence of PA-6.

Whole cell patch clamp measurements were done as described before [28] using an AxoPatch 200B amplifier controlled by pClamp9 software (Molecular devices, Sunnyvale, CA, USA) at 21 °C. Whole cell I_KIR2.1_ measurements were performed by applying 1 s test pulses ranging between −120 and +30 mV, in 10 mV increments, from a holding potential of −40 mV, and with series resistance compensation of at least 70%. Steady state current at the end of the pulse was normalized to cell capacitance and plotted versus test potential (corrected for liquid junction potential). Extracellular solution contained 140 mM NaCl, 5 mM KCl, 1 mM CaCl_2_, 1 mM MgCl_2_, 6 mM glucose, 17.5 mM NaHCO_3_, 15 mM HEPES, pH 7.4/NaOH. Pipette solution contained 125 mM potassium gluconate, 10 mM KCl, 5 mM HEPES, 5 mM EGTA, 2 mM MgCl_2_, 0.6 mM CaCl_2_, 4 mM Na_2_ATP, pH 7.20/KOH.

**Fig. 1:**
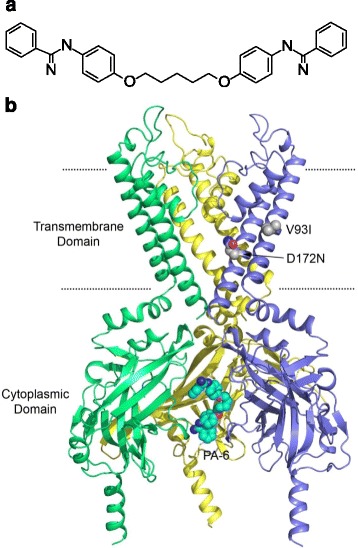
**a** Chemical structure of PA-6 (drawn in Accelrys Draw 4.1). **b** Compound PA-6 docked into a homology model of K_IR_2.1. Three subunits are shown in side view; the fourth is removed for clarity. The location of mutations V93I (M1 helix) and D172N (M2 helix) are depicted in spheres representation. PA-6, bound to the cytoplasmic domain, is shown as cyan spheres. Nitrogen atoms are coloured blue, oxygen atoms are shown in red. Approximate membrane boundaries are indicated as black dotted lines. The figure was generated with Pymol

PA-6 (Fig. 1a) was custom synthesized by Endotherm GmbH (Saarbrücken, Germany).

### Western blot

HEK293T cells were grown in 60 mm culture dishes containing 3 mL DMEM supplemented with 10% FCS, L-Glutamine and Pen-Strep, and transfected using linear polyethylenimine (PEI) (PolysciencesInc, Eppelheim, Germany) as described earlier [29]. Cell lysates were prepared in buffer D (20 mM HEPES, 125 mM NaCl, 10% glycerol, 1 mM EDTA, 1 mM EGTA, 1 mM dithiothreitol, and 1% Triton X-100, pH 7.6, supplemented with 1 mM PMSF and 10 μg/mL aprotinin). 20 μg protein lysate was separated by 10% SDS-PAGE and blotted onto nitrocellulose membrane. Ponceau staining was used to reveal equal protein loading and subsequent quantification. Blots were blocked with 5% (*v*/v) chicken egg yolk in TBST (20 mM Tris-HCl pH 8.0, 150 mM NaCl, 0.05% (*v*/v) Tween-20) for 1 h at room temperature. K_IR_2.1 WT and mutants were detected by N-terminal K_IR_2.1 antibody (Santa Cruz Biotechnology, Dallas Tx, USA, cat. No. sc-18708) and peroxidase-conjugated donkey anti-goat secondary antibody (Jackson ImmunoResearch, West Grove, PA, cat. No. 705-035-003) followed by ECL detection procedure (GE Healthcare, Hoevelaken, The Netherlands). For quantification purposes, untransfected HEK293T cells were used as blank and protein levels were normalized to ponceau staining levels. Differences between group averages were tested by using a one-way ANOVA with a Bonferroni’s post-hoc test.

### Immunofluorescence microscopy

HEK293T and MES-1 cells were cultured on pre-coated (0.1% gelatin) glass coverslips and transfected using Lipofectamin (Invitrogen, Breda, The Netherlands). Cell fixation, immunolabeling and imaging were performed exactly as described earlier [29]. Antibodies used were anti-K_IR_2.l (1:250; Santa Cruz Biotechnology, cat. no. sc-18708) and anti-Pan Cadherin (1:800, Sigma-Aldrich, St. Louis MO, USA, cat. No. C1821). HEK293T cells were imaged by confocal microscopy using a Zeiss LSM 700 confocal microscope (Carl Zeiss Microscopy GmbH, Germany) equipped with a 63× oil immersion objective (NA 1.4). Excitation was performed with an air-cooled Argon ion laser (LASOS, RMC 7812Z, 488 nm) for GFP and a HeNE (LASOS, SAN 7450A, 543 nm) laser for DyLight. MES-1 cells were imaged using a conventional Nikon eclipse 80i light microscopy equipped with a 40× objective (NA 0.75).

## Results

### V93I and D172N mutations do not interfere with the PA-6 K_IR_2.1 channel interaction

To determine whether V93I or D172N mutations in the K_IR_2.1 channel may interfere with PA-6 current block, mutations and PA-6 channel interaction were modelled. Figure 1b shows the docking result of PA-6 into the V93I/D172N double mutant homology model of K_IR_2.1. The results demonstrate that these mutations are unlikely to influence binding of PA-6 to the previously identified binding site in the cytoplasmic domain [20]. Both mutations are located in the transmembrane domain and thus >30 Å away from the binding site.

### PA-6 inhibits inward rectifier currents carried by V93I and D172N mutant K_IR_2.1 channels

**Fig. 2:**
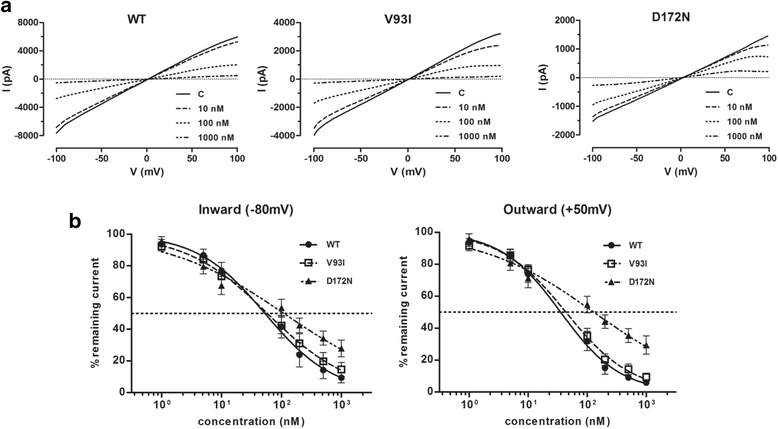
PA-6 dose dependently inhibits I_K1_ currents carried by homotypic wild-type (WT), V93I and D172N K_IR_2.1 channels measured in the inside out mode. **a** Steady state K_IR_2.1 current traces from WT, V93I and D172N K_IR_2.1 channel containing inside-out patches elicited by a voltage ramp protocol from −100 to 100 mV, under baseline conditions (C) and upon application of 10, 100 and 1000 nM of PA-6. Measurements were performed using symmetrical high potassium concentrations at both sides of the patch. **b** IC_50_ curves of PA-6 for the inward (at −80 mV) and outward (at +50 mV) K_IR_2.1 currents of WT (*n* = 10), V93I (*n* = 9) and D172N (*n* = 11) channel containing patches. Error bars indicate s.e.m

To determine the functional effects of PA-6 on the K_IR_2.1 gain-of-function mutant channels, expression constructs were transiently transfected into HEK293T cells. Currents were measured in the inside-out mode, to allow for direct access of PA-6 to the cytoplasmic channel pore, of the patch-clamp technique using a ramp-protocol from −100 to +100 mV. PA-6 dose dependently inhibited inward rectifier inward (at −80 mV) and outward (at +50 mV) currents carried by WT, V93I and D172N K_IR_2.1 channels (Fig. 2a). A small voltage dependency of block was observed for WT and both mutant channels as reflected in small, but not significant, changes in IC_50_ values obtained at −80 and +50 mV for each K_IR_2.1 type. Interestingly, in contrast to WT and V93I, D172N outward current was less sensitive for PA-6 block than inward current. Whereas WT and V93I displayed virtually identical dose dependent block (IC_50_ of 52.9 and 58.0 nM at −80 mV and 35.5 and 43.6 nM at +50 mV for WT and V93I channels respectively), D172N channels were approximately two to three fold less sensitive (IC_50_ 109.3 nM at −80 mV and 128.9 nM at +50 mV) (Fig. 2b).

**Fig. 3:**
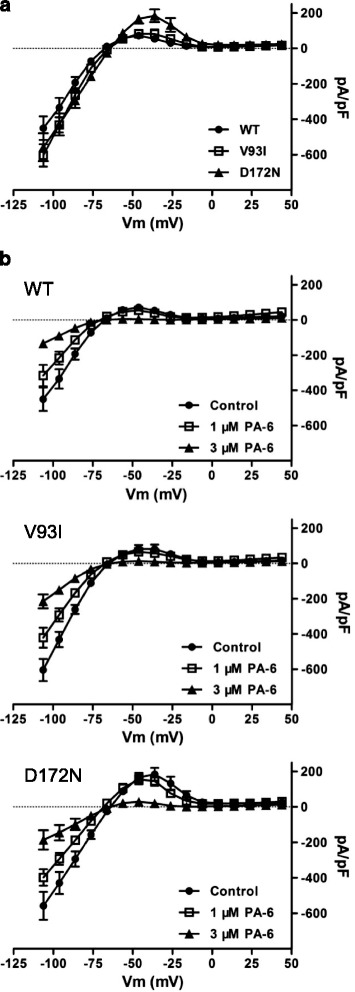
PA-6 dose dependently inhibits I_K1_ currents carried by homotypic wild-type (WT), V93I and D172N K_IR_2.1 channels in the whole cell mode. **a** Comparison of whole cell IV-curves obtained from HEK293 cells expressing WT (*closed circles*), V93I (*open squares*) or D172N (*closed triangles*) K_IR_2.1 channels. Currents were elicited by 1 s test pulses ranging between −120 and +30 mV, in 10 mV increments and correct for liquid junction potential. **b** Whole cell steady state current IV-curves from WT, V93I and D172N K_IR_2.1 channels expressed in HEK293 under control (*closed circles*), 1 μM PA-6 (*open squares*) and 3 μM PA-6 conditions. Voltage protocol as in panel **a**

Using the whole cell mode of the patch clamp technique, gain-of-function of D172N mutant became apparent as a change in the rectification index ((1-(outward current at −40 mV divided by inward current at −100 mV)) [30], not correct for liquid junction potential) from 0.83 ± 0.11 (*n* = 9, mean ± s.d.) for WT channels to 0.70 ± 0.06 (*n* = 10, *P* < 0.01) in D172N channels (Fig. 3a), in accordance with earlier findings [2]. No change in rectification index of V93I (0.87 ± 0.09, *n* = 10, n.s.) was found. In addition, whereas PA-6 sensitivity was decreased in the whole cell mode, compared to the inside-out mode no differences were observed between WT and mutant channels (Fig. 3b). In the presence of 1 μM of PA-6, a non-significant (n.s.) reduction of outward I_K1_ at −50 mV by 28 ± 36%, 18 ± 20% and 10 ± 6%, for WT, V93I and D172N channels respectively was observed. At 3 μM, PA-6 significantly inhibited I_K1_ at −50 mV by 94 ± 6% (*P* < 0.05), 77 ± 29% (*P* < 0.05) and 86 ± 7% (*P* < 0.01), for WT, V93I and D172N channels, respectively. For the inward current at −110 mV, 1 and 3 μM PA-6 inhibited I_K1_ by 30 ± 16% (n.s.) and 68 ± 15% (*P* < 0.01), 28 ± 14% (*P* < 0.05) and 64 ± 4% (*P* < 0.001), 30 ± 10% (n.s.) and 83 ± 1% (*P* < 0.01) for WT, V93I and D172N channels, respectively. We conclude that in the whole cell mode, PA-6 inhibits WT, V93I and D172N channels with similar efficacy resulting in IC_50_ values between 1 and 3 μM.

### V93I and D172N mutations do not affect PA-6 mediated increase in K_IR_2.1 expression and intracellular accumulation

**Fig. 4:**
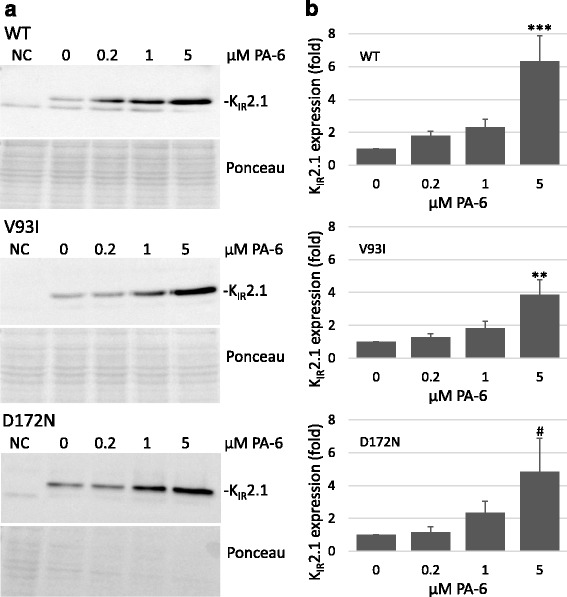
PA-6 application for 24 h increases WT, V93I and D172N K_IR_2.1 channel expression. **a** Dose-dependent (0-5 μM) effect of 24 h exposure to PA-6 on HEK293T cells transiently transfected with WT, V93I and D172N K_IR_2.1 channels. NT indicates non-transfected cells. Ponceau staining was used as loading control for quantification (**b**) Summarized quantification data from WT (*n* = 11 independent western blots), V93I (*n* = 11) and D172N (*n* = 13) are shown in mean ± s.e.m. *** *P* < 0.001 vs. control, ** *P* < 0.01 vs. control, #*P* = 0.09 vs. control

When applied at concentrations of 10 μM, PA-6 is able to enhance expression of WT K_IR_2.1 in cells stably expressing GFP-tagged K_IR_2.1 [20]. To assess the effects of PA-6 on expression of WT and mutant channels in transiently transfected HEK293T cells, cultures were treated with 0, 0.2, 1 and 5 μM PA-6 for 24 h after which expression levels were detected by Western blot analysis. PA-6 treatment increased K_IR_2.1 expression levels for all three variants (Fig. 4a). Strongest responses were obtained with 5 μM PA-6 added to the medium that reached significance for WT (*P* < 0.001) and V931 (*P* < 0.01) whereas a trend was observed for D172N (*P* = 0.09) (Fig. 4b). Lower concentrations of PA-6 did not result in significant increased expression.

**Fig. 5:**
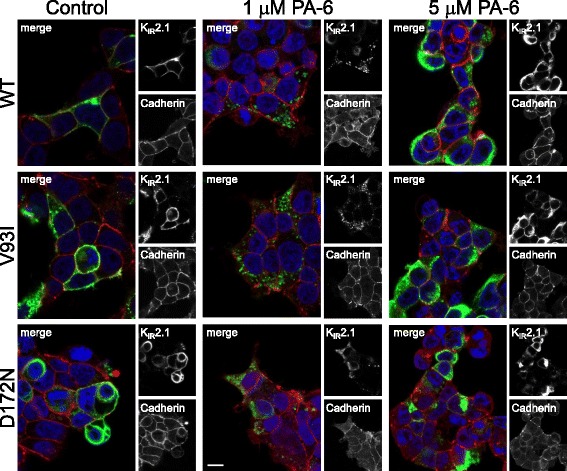
PA-6 treatment for 24 h induces intracellular K_IR_2.1 accumulation of WT, V93I and D172N channel proteins in HEK293T cells. Confocal microscopy optical slices (0.7-0.8 μm) displaying K_IR_2.1 (*green*) localization in HEK293T cells transfected with WT, V93A and D172N channels under control conditions and following 24 h of PA-6 (1 and 5 μM). Cadherin (*red*) co-staining identifies the position of the plasma membrane. DAPI (*blue*) is used to visualize nuclei. Scale bars represent 10 μm

**Fig. 6:**
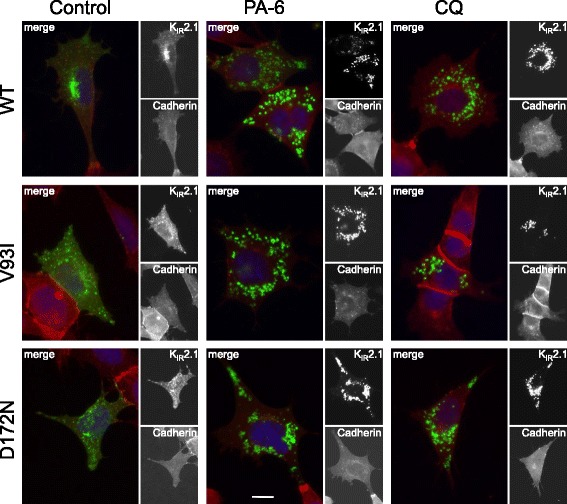
PA-6 treatment for 24 h induces intracellular K_IR_2.1 accumulation of WT, V93I and D172N channel proteins in MES-1 cells. Whole cell K_IR_2.1 (*green*) and Cadherin (*red*) localization in MES-1 cells transfected with WT, V93I or D172N channels in non-treated (control), PA-6 (5 μM, 24 h) or chloroquine (10 μM, 24 h) conditions imaged by conventional fluorescent microscopy. DAPI (*blue*) was used to identify nuclei. Scale bars represent 10 μm

To detect the subcellular location of the WT, V93I and D172N channels following PA-6 application, confocal immunofluorescent microscopy was performed in HEK293T cells. In non-treated control cells, both WT and mutant channels were mainly expressed at the plasma membrane, where they demonstrate colocalization with Cadherin (Fig. 5 left column), whereas PA-6 treatment (1 μM) for 24 h induced an intracellular increase of the channel proteins in small aggregates, irrespectively whether these are WT or mutant (Fig. 5, middle column). PA-6 treatment at 5 μM for 24 further increased the level of intracellular accumulation (Fig. 5, right column). To exclude any potential effect of cell-type specific response, immune detection was also performed in the mesoderm-like cell line MES-1 as shown previously [29] which yielded similar responses (Fig. 6). In control cells, WT and mutant channels were localized mainly at the plasma membrane, whereas 5 μM of PA-6 (24 h) resulted in intracellular accumulation, in a fashion similar as for chloroquine although the latter treatment resulted in aggregates more equally in size [27, 31].

## Discussion

Due to the high risk of sudden cardiac death, ICD implantation is indicated in congenital SQT patients. However, pharmacological treatment is warranted in young children not amenable for ICD implantation, in patients refusing an ICD and as a bridge to ICD therapy [10]. In congenital SQT patients, responsiveness of the QTc interval towards class IC and class III antiarrhythmics were unsatisfactory. SQT patients did not show anticipated responses to flecainide, d-sotalol or ibutilide [32, 33]. Only hydroquinidine was able to prolong QTc to borderline or normal duration [32, 33]. Short QT syndrome type 1 (SQT1) results from gain-of-function mutations in the K_v_11.1 (hERG) channel encoded by the *KCNH2* gene, and is the best studied SQT subtype with respect to pharmacological treatment. The N588K gain-of-function mutation appears a hotspot in SQT1. Interestingly, N558K channels were less sensitive for Class III antiarrhythmics like d-sotalol [34], and E-4031 (11-fold) [11]. Accordingly, d-sotalol was unable to prolong the QT interval in SQT1 N558K patients [34]. In contrast, disopyramide (1.5-fold) and hydroquinidine (3.5-fold) displayed smaller differences in IC_50_ values for WT and N558K K_v_11.1 channels, respectively. Clinical studies indeed showed favourable responses to hydroquinidine in SQT1 [33, 35], whereas QTc prolongation in non-K_v_11.1 SQT patients was smaller [33]. The SQT2 associated mutation V307L in the K_v_7.1 channel was shown to be equally sensitive for mefloquine as its WT variant, on which basis the authors suggested that this drug may be an effective treatment strategy in this patient population [14]. Interestingly, the same V307L mutation increased the IC_50_ value for the K_v_7.1 inhibitor Chromanol293B by 7-fold [36], indicating again that a mutation specific pharmacological approach is favourable. The SQT3 associated D172N mutation in K_IR_2.1 was equally sensitive for chloroquine (1.2-3.3 μM) as its WT counterpart (1.4-2.4 μM) measured in the whole cell mode [12, 13]. Here we demonstrate that upon acute superfusion, PA-6 is also able to inhibit D172N mutant K_IR_2.1 channel with an IC_50_ only two to three fold higher than that of WT channels, measured in the inside-out mode, and that potency of inhibition of the V93I channel was similar as for WT. In the whole cell mode, acute PA-6 superfusion inhibits WT, V93I and D172N with similar efficacy. Therefore, PA-6 could potentially be effective in addressing SQT3 and AF associated with each of these two mutations.

In contrast to PA-6, chloroquine suffers from lack of specificity as it significantly inhibits delayed rectifier (I_Kr_), sodium (I_Na_) and l-type calcium (I_Ca-l_) currents [20, 37]. Upon long-term exposure however, both chloroquine and PA-6 are able to increase K_IR_2.1 channel expression [20, 27, 31, 38]. However, due to its lower potency, the concentrations at which chloroquine affected trafficking (5 μM) are slightly closer to its IC_50_ for acute blockade than seen for PA-6. Furthermore, the majority of PA-6 induced increase of K_IR_2.1 channel expression as detected by Western blot upon chronic exposure results from intracellular accumulation instead of functional channels at the plasma membrane.

The acute channel inhibiting effect of a drug may or may be not be correlated with its chronic effect on ion channel expression. For example, pentamidine inhibits the K_IR_2.1 channel acutely and decreases K_IR_2.1 expression upon chronic treatment [38, 39], but its structural derivative PA-6 inhibits K_IR_2.1 currents acutely while it increases channel expression chronically as shown here and previously [20]. In the former case, both drug effects are additive and maybe synergistic. In the case of PA-6, acute and chronic effects are opposite, but at 1 and 3 μM, the acute effects on current inhibition are stronger than the increases in channel expression levels upon chronic exposure. Only upon acute termination of PA-6 application, the effects on expression may temporarily prevail over the acute blocking effect (or absence thereof) resulting in enhanced I_K1_.

## Limitations

The effects of PA-6 on WT, V93I and D172N K_IR_2.1 channels have only been tested in ectopic expression systems and therefore the effects of their blockade on cardiac action potential characteristics could not be evaluated.

## Conclusions

In the K_IR_2.1 ion channel, V93I and D172N gain-of-function mutations do not blunt the inhibitory capacity of PA-6. PA-6 application results in enhanced K_IR_2.1 protein expression, mainly localized in intracellular aggregates. From our findings presented here, we conclude that PA-6 may be considered for further preclinical evaluation for treatment of congenital SQT3 and AF.

## Abbreviations

AF: Atrial fibrillation
GFP: Green fluorescent protein
HEK: Human embryonal kidney
I_Ca-l_: L-type Calcium current
ICD: Implantable cardioverter-defibrillator
I_K1_: Inward rectifier potassium current
I_KACh_: Acetylcholine activated inward rectifier potassium current
I_Kr_: Delayed rectifier current
I_Na_: Sodium current
PA-6: Pentamidine analogue-6
SQTx: Short QT syndrome type x
WT: Wild-type
